# Nonlinear growth: an origin of hub organization in complex networks

**DOI:** 10.1098/rsos.160691

**Published:** 2017-03-22

**Authors:** Roman Bauer, Marcus Kaiser

**Affiliations:** 1 Institute of Neuroscience, Newcastle University, Newcastle upon Tyne NE2 4HH, UK; 2 Interdisciplinary Computing and Complex BioSystems Research Group (ICOS), School of Computing Science, Newcastle University, Newcastle upon Tyne NE1 7RU, UK

**Keywords:** complex networks, hubs, neural development, physical space, rich-club organization, nonlinear growth

## Abstract

Many real-world networks contain highly connected nodes called hubs. Hubs are often crucial for network function and spreading dynamics. However, classical models of how hubs originate during network development unrealistically assume that new nodes attain information about the connectivity (for example the degree) of existing nodes. Here, we introduce hub formation through nonlinear growth where the number of nodes generated at each stage increases over time and new nodes form connections independent of target node features. Our model reproduces variation in number of connections, hub occurrence time, and rich-club organization of networks ranging from protein–protein, neuronal and fibre tract brain networks to airline networks. Moreover, nonlinear growth gives a more generic representation of these networks compared with previous preferential attachment or duplication–divergence models. Overall, hub creation through nonlinear network expansion can serve as a benchmark model for studying the development of many real-world networks.

## 1. Introduction

### 1.1. Background

One of the most commonly used network growth models that generate hubs [1,2], which often are essential for network function [3,4] and spreading dynamics [5], is the preferential attachment model (‘rich gets richer’) [6]. However, it assumes that connections of a new node form depending on how well connected a potential target node is compared to all other existing nodes. The knowledge of such global information is often difficult to justify in a biological context; for example, axonal growth cones in neuronal networks can only sense their local environment. It is unclear whether and how the preferential attachment model can be applied to such network growth dynamics, as it often cannot provide a mechanistic explanation. The duplication–divergence model [7], which is inspired by the evolution of protein–protein interaction (PPI) networks, on the other hand, needs a duplication of previously existing connection patterns that is unlikely for many non-biological networks (e.g. flight connections between airports). Moreover, the divergence step entails a non-trivial coordination between duplicated nodes (i.e. a new connection is pruned with a pre-specified probability, but only if its counterpart is not). Such communication is difficult to justify in many biological networks, where the information exchange between maturing connections is limited.

### 1.2. The principle of nonlinear growth

We propose a novel model that is based on exponential network growth. It assumes that the number of new nodes increases nonlinearly with time, which is a general principle of growth and development: systems ranging from the worldwide web to neural networks in the brain experience episodes of exponential growth where the number of new nodes at one time will be larger than the number of new nodes at a previous time. As an example for growth in biological systems, the number of cells per unit volume (*c*) in the growth phase of bacterial cultures can be described by an exponential function: *c*(*n*)=*c*_1_⋅2^*n*^, where *c*_1_ is the initial unit volume of the culture, and *n* is the number of divisions a cell has undergone [8]. For brain evolution, it was proposed that new neural structures form by separation of already existing areas [9], with the number of brain areas then increasing exponentially. The early growth of the Internet [10] would be an example for non-biological systems.

## 2. Material and methods

### 2.1. Data analysis

**Figure 1.**
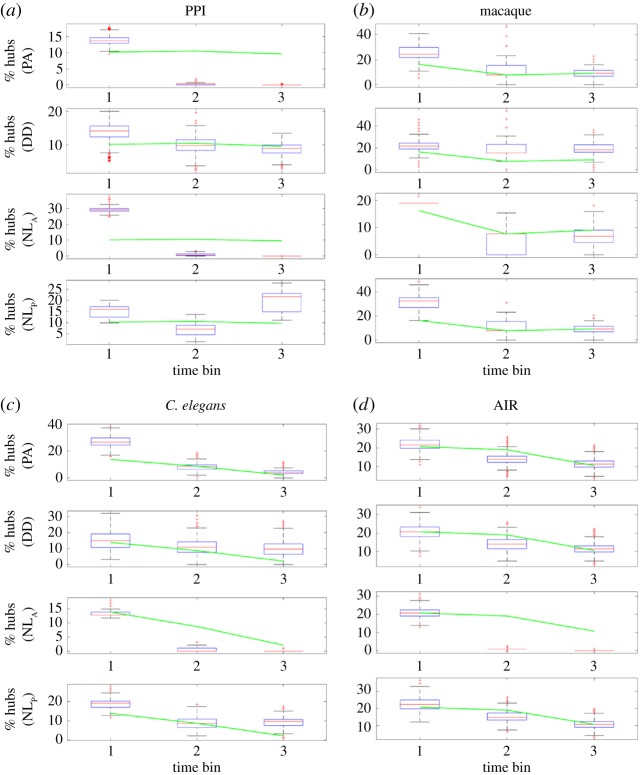
Trajectories of hub occurrences for the PPI (*a*), macaque (*b*), *C. elegans* (*c*) and AIR (*d*) datasets. Green lines indicate the experimental data, while the box-plots show the results of the (optimized) model-generated samples. The overall performance of the models is summarized in table 1. The green lines are identical for the same datasets, but are displayed within different axes.

Analysis and implementation of growth models were conducted using Matlab R2014b (Mathworks Inc.). Visualization of the hub occurrences and node degrees across development (figure 1; electronic supplementary material, figure S4) was done by binning the maturation times, i.e. the times of nodes formation during network development. For the *Caenorhabditis elegans* and AIR datasets, three maturation bins (columns) were computed such that the number of nodes in each bin were approximately the same. For the PPI and macaque datasets, three age categories were assigned, and so the number of maturation bins is matched accordingly.

**Figure 2.**
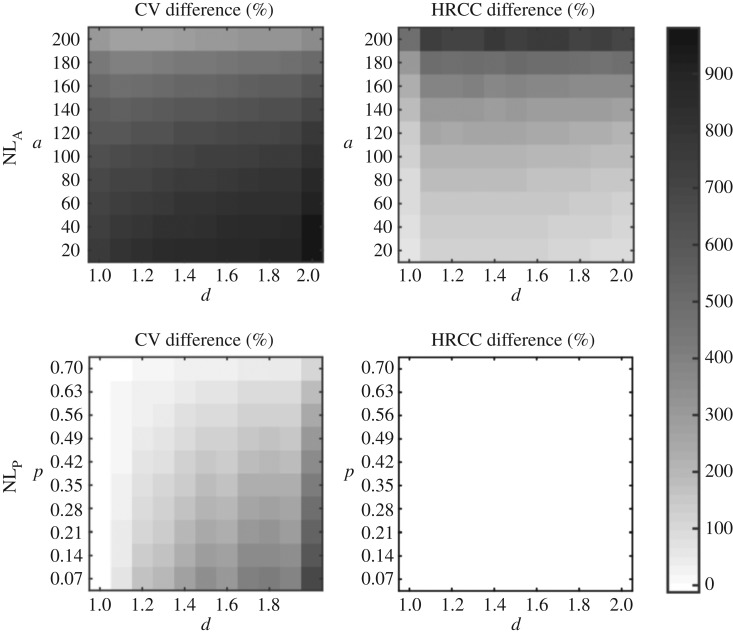
Influence of model parameters on degree variation (CV) and mean rich-club coefficient between hubs (HRCC) for the *NL*_A_ and the *NL*_P_ models, across different model parameters. The greyscale heatmap displays relative differences from CV and HRCC values of regular, random networks with the same number of nodes and edges. Darker shadings indicates higher CV and HRCC values, respectively, than expected from the control networks. Overall, the figure demonstrates that nonlinear growth can produce hub-related complex network properties. In contrast with the *NL*_A_ model, the *NL*_P_ model does not generate networks where hubs preferentially connect with each other (for all parameter constellations shown, a Wilcoxon rank sum test at the 5% significance level failed to reject the null hypothesis that the deviations have zero median). This is because the early-born nodes are equally as likely to connect to other early-born nodes as to later-born nodes, because the connection probability is fixed throughout network growth. Analogous results for the PA and DD models and larger networks are shown in the electronic supplementary material, figure S2.

The results shown in figure 2 and the electronic supplementary material, figure S2A are computed based on 200 networks for each of the parameter sets. Random, regular networks of equal size were created as follows: first, the mean edge density of these nonlinearly grown networks was computed. Then, the corresponding probability of edge formation in random, regular network growth was computed (this regular growth corresponds to the *NL*_P_ model with parameter *d*=1). Using this probability, 200 regular, linearly grown networks were generated for each parameter set. Finally, the corresponding coefficient of variation (CV) and hub-rich-club coefficient (HRCC) values were computed, which served as the comparative values for the shown relative deviations. Owing to high demands in computer resources, the results shown in the electronic supplementary material, figure S2B are based on 20 networks.

For the assessment of the model performances, 1000 network samples were generated for each analysed network measure (CV value, HRCC measure and the maturational pattern of hub occurrence). Network samples where the number of edges deviated by more than 10% from the target number were replaced by newly generated samples matching this criterion. For the CV and HRCC values, a model was classified as explanatory if a statistical test is passed for the experimental value to stem from the model-generated distribution, using the z-statistic with a significance of 1%, or if the value derived from the respective dataset was within the model-generated interval. For the maturational trajectories, the same approach was taken for the percentage of hubs in each maturation bin. A model was classified as explanatory if at least one of the model-generated numbers of hubs matched the observed value, or if the model-generated distribution passed the *z*-test for the observed value.

Rich-club coefficients and complementary network measures (clustering coefficient *C*, modularity index *Q* and characteristic path length *L*) were computed using the Matlab scripts obtained from the Brain Connectivity Toolbox [11] (https://sites.google.com/site/bctnet/measures/list). Degree preserving reference networks were computed using the *randmio_und.m* function (also from the Brain Connectivity Toolbox).

### 2.2. Parameter optimization

We conducted parameter optimization using simulated annealing, such that the model-generated networks exhibit measures as close as possible to the empirical values. The Matlab function simulannealbnd.m was used for conducting simulated annealing. The cost function was defined as: *A*⋅*r*_1_+*r*_2_. *r*_1_ and *r*_2_ are the relative errors of the number of edges and the CV or HRCC values, respectively. *A* is a penalty factor, and was manually adjusted in case the generated mean number of edges deviated largely from the empirical value (electronic supplementary material, figure S3). For the assessment of the models’ performance, model-generated networks where the number of edges was beyond 10% of the dataset’s edges were discarded. The resulting distribution of the number of edges had to pass a test using the *z*-statistic on the 1% significance level. In total, 1000 networks were generated for the visualizations of the CV values, the HRCC measures and the percentage of hub nodes.

Simulated annealing was also used for optimizing model parameters to yield the percentages of hubs in the three maturation bins. This was done under the null hypothesis that the observed percentage of hubs in a particular maturation bin stems from the model-generated distribution. The probability for observing the measured percentage under this null-hypothesis, as obtained from a *z*-test, was maximized in order to obtain the model parameters.

As the PPI and AIR datasets are bidirectional, we implemented that the formation of a connection in one direction also entails the connection into the other. Therefore, the target number of edges used in the optimization process was half the actual edges. The model-generated connectivity matrices were added to their transposed form, such that the resulting matrix was symmetrical and ideally matched the actual number of edges of the two datasets. For the assessments with regard to the macaque and *C. elegans* datasets, the model-generated networks were generated by probabilistically adding bidirectional connections, in order to match the proportion of unidirectional versus bidirectional connections derived from the datasets.

## 3. Results

### 3.1. Datasets

**Figure 3.**
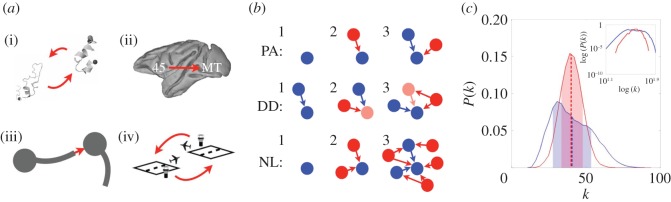
Datasets and models. (*a*) Types of connections in real-world networks: (i) interactions between proteins, (ii) connections between brain regions in the macaque, (iii) connections using chemical synapses, in *C. elegans*, and (iv) flight connections between airports. Note that connections for (ii) and (iii) may be unidirectional. (*b*) Growth models leading to highly connected nodes. PA, preferential attachment [6]: new nodes (red) preferentially connect to nodes with higher degrees. DD, duplication–divergence model: at each step, a random node (light red) is duplicated (red) together with its links. NL, nonlinear growth: the number of new nodes that are added at each step increases nonlinearly over time. New nodes project to already present nodes (blue) establishing on average *a* connections (NL_*A*_) or link to each existing node with a probability *p* (NL_P_). (*c*) Exemplary distributions for node degree *k* (inset: log–log plot) for networks from linear (red) and nonlinear growth (blue). Linear growth corresponds to the scenario where the network size increases linearly, i.e. only one node is added at each time step. Shaded areas show the standard deviation around the mean degree (dashed line). Nonlinear growth yields a wider distribution with more hubs.

In order to compare the explanatory power of the nonlinear growth model with other models of network growth, we analysed four datasets including longitudinal information (figure 3*a*). The collected datasets of evolving networks include (i) the protein–protein interaction network in yeast [12], (ii) axonal connections between brain regions in the rhesus monkey (macaque) [13], (iii) the network between neurons in the nematode *C. elegans* [2,14] and (iv) the network of flights between international airports worldwide [15]. The maturation time of a node (i.e. the time when the node is added to the growing network) is defined as (i) the time during evolution when a protein first occurs, (ii) the maturation time of a brain region during development, (iii) the birth time of a neuron and (iv) the year when an airport was established, respectively (cf. table 2). The datasets (ii) and (iv) were collected by us and are therefore novel. All the used datasets are described in more detail in the electronic supplementary material.

### 3.2. The nonlinear growth model

The nonlinear growth model (NL) assumes that the network size increases nonlinearly/exponentially with time. At each developmental stage *t*, the network expands by *d*^*t*^ nodes (*d* is a parameter of the model) until a given network size is reached (figure 3*b*). The (rounded) number of newly formed nodes project to the already present nodes. Importantly, in our model the formation of connections does not rely on any properties of the nodes. Hence, a newly developed node does not need to check whether its target node fulfils specific conditions, but forms connections dependent upon a model parameter that is initially specified. NL substantially differs from the well-known Erdös–Rényi model (ER) [16], where the number of connections increases linearly, but the nodes are initially present already. As demonstrated in [17], linear network growth (i.e. addition of one node at a time), in addition to ER-like formation of connections, can yield complex network features such as small-worldness and scale-free degree distribution. Hence, the addition of new nodes is a crucial factor in network growth models. In this study, we show that also the dynamical nature of node addition strongly shapes the resulting network structures.

We distinguish two different scenarios of nonlinear growth. In the first case (NL_A_), the number of target nodes that a new node projects to is computed as follows: a random value is drawn from a normal distribution with mean *a* and standard deviation *a*/2, where *a* indicates a model parameter. This value is rounded and limited by the cut-off points 0 and the current number of nodes in the network. Hence, the number of projections that a new node makes stays approximately the same throughout development. In a second scenario (NL_P_), new nodes connect to any existing node with probability *p*, so that the number of a new node’s outgoing projections is proportional to the number of existing nodes. In this scenario, the number of outgoing connections per node will increase during development, which is in contrast with the NL_A_ scenario. A simplified, algorithmic description of nonlinear growth and its two versions is shown in algorithm 1. As the NL model does not require any knowledge of a global variable, both scenarios are based solely on local information.


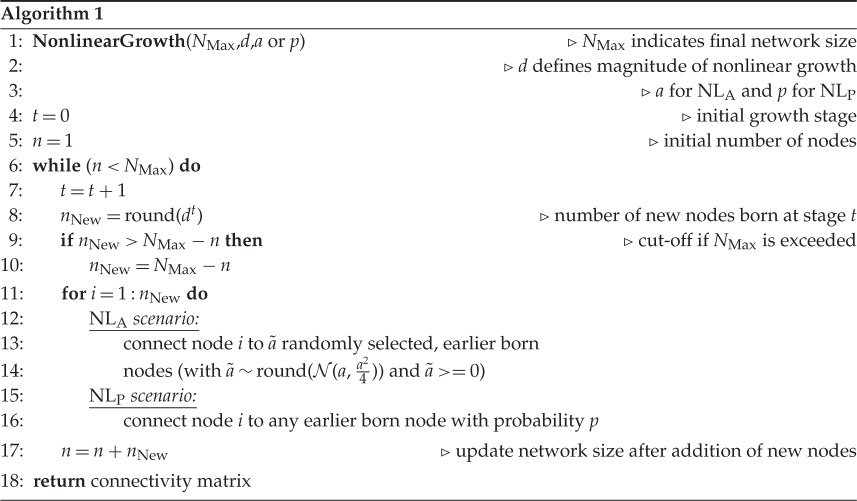


### 3.3. Alternative models

We have compared the NL model with the extended preferential attachment (PA) and the duplication–divergence (DD) models (figure 3*b*). For the classical preferential attachment model [6], at each time step a single node is added to the network and forms connections with existing nodes following a preference for highly connected nodes (i.e. hubs). For simplicity, our PA growth model starts from a single node (we confirmed that this initial condition yields scale-free degree distributions). The first parameter *p*_1_ of the PA model determines to how many nodes the newly added node connects on average. A second parameter *p*_2_ of this extended version specifies a baseline probability to connect to any of the nodes in the network, independent of their degree. The probability that a newly added node connects to node *i* of the network is then computed as $p_{i}=p_{1}(k_{i}/\sum_{j}k_{j})+p_{2}$. The PA model can be understood as an extension of a linear network growth model, where newly added nodes have knowledge about target nodes.

The DD model is implemented as described in [7]. Each time step consists of a duplication and a divergence step. In the duplication step, a random node is duplicated, i.e. it projects to the same connection partners as the original node. The connection between the two duplicated nodes prevails with probability *p*. In the subsequent divergence step, one of the two connections of the partners with the duplicated nodes is pruned with probability *q*. Maximally one of the two connections can be pruned, and so this model is based upon non-trivial coordination between the two connections. To allow for a fair comparison, both the alternative and the nonlinear growth models are based on two parameters.

### 3.4. Analysis of model-generated networks

We have assessed the networks’ range of degrees through the coefficient of degree variation (CV), which is the standard deviation divided by the mean. For a linearly growing network with random formation of connections, all degrees are relatively close to the average degree, while for networks that contain highly connected nodes, the standard deviation of the node degree is larger due to such outlier nodes (figure 3*c*; electronic supplementary material, figure S1). We find that CV value distributions of model-generated networks are sufficiently narrow to allow to differentiate the models from each other, and so are well suited for a comparison of the different models. Moreover, because this study focuses on the development of hub-related network organization, we did not investigate other metrics of the degree distribution that are not directly related to hubs.

Complementary to the degree distribution, we also investigated the rich-club organization, which captures the tendency for hubs to interconnect with one another, rather than with low-degree nodes [18]. In many real-world networks, such as the neuronal connectivity of *C. elegans* [19] and the inter-areal projections in cat [20], macaque [21] or human [22], hub nodes connect with each other more strongly than with nodes of lower degree. Conversely, it has been shown that protein interaction networks do not contain rich clubs [18]. To quantify this tendency for connectivity among hubs, we used the rich-club coefficient [18] defined as *ϕ*(*k*)=2*E*_>*k*_/*N*_>*k*_(*N*_>*k*_−1), where *E*_>*k*_ is the number of edges among the *N*_>*k*_ nodes that have a higher degree than *k*. Essentially, this coefficient measures the ratio of existing connections to the potential number of connections among a given set of nodes. Hence, the rich-club coefficient is a vector of values (different coefficients for different values of *k*). In order to simplify the comparison between different complex networks, we summarize the rich-club organization as the mean of the rich-club coefficients for all degrees *k* that qualify a node as being a hub. We define this so-called HRCC as: $\mathrm{HRCC}=\sum_{k\in \Gamma }\phi (k)/|\Gamma |$, where *Γ* is the set of the degrees of all hub nodes in a given network and |*Γ*| is the number of elements in this set. We classify a node as a hub if the number of its connections *k* is at least one standard deviation above the average number of connections of a node (*k*≥*μ*+*σ*). Notably, rich-club organization is different from the assortativity property [18], and is a particularly well-esteemed measure in many biological networks.

Figure 2 and the electronic supplementary material, figure S2 visualize that nonlinear growth can indeed produce networks with CV and HRCC values that are much larger than those of regular, random networks across different parameter values. As expected, these hub-related network measures are very dependent on the two model parameters: the nonlinear growth exponent *d* strongly affects CV values, by increasing the spread of the degree distribution. The *NL*_P_ model yields, due to equal probability of connections among all nodes, no rich-club organization. However, for *d*>1 it produces hubs across a wide spectrum of parameters. For the *NL*_A_ model, the CV values and the rich-club organization express themselves much beyond what is expected based on comparable, regular networks (note also the sudden increase of HRCC values for *d*>1). Detailed and asymptotic behaviour of the network measures for the *NL*_P_ model are consistent across different network sizes (electronic supplementary material, figure S2). In addition to the CV and HRCC measures, the hub nodes were additionally analysed for their developmental time, and the models were assessed for their explanatory power with regard to this temporal aspect (figure 1).

**Figure 4.**
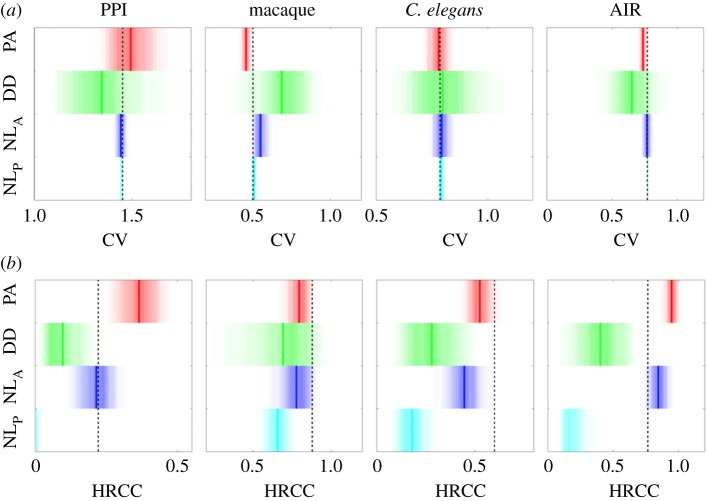
Generation of network properties. Degree variation (CV, *a*) and mean rich-club coefficient between hubs (HRCC, *b*) of the models in relation to values of real-world networks (black dashed lines). Red, green, blue and cyan shaded regions indicate the distribution of values that PA, DD, NL_A_ and NL_P_ could yield, respectively. Thick coloured lines indicate the mean values of the distributions. The respective, model-generated connection numbers are displayed in the electronic supplementary material, figure S3.

We find that the analysed models reproduce many properties of the collected networks; preferential attachment matches some of the network measures (figure 4) but, for example, fails to generate the same number of connections when producing the CV value of the airline network (electronic supplementary material, figure S3). DD and NL models match both CV and number of connections. While the NL_P_ can account for all the CV values and the maturational trajectories, it cannot yield the HRCC values. Hub nodes are predominantly established early on [2,23] and show a distinct pattern of creation over time (electronic supplementary material, figure S4). Other relevant developmental properties are shown in the electronic supplementary material, figure S5.

Our results demonstrate that only the NL_A_ model can account for all the datasets’ CV and HRCC values. It provides an intuitive explanation for the origins of rich-club connectivity; during early network development, there are not many nodes to project to, therefore most early born nodes project to each other. As early developing nodes are likely to become highly connected, hubs are predominantly connected among each other. Note, however, that nonlinear growth is also crucial: even a modified PA model, where the number of outgoing connections is also limited, cannot reproduce CV and HRCC features (electronic supplementary material, table S1; see electronic supplementary material, figures S6 and S7 for other features).

In summary, only nonlinear growth can reproduce the analysed features of real-world networks (table 1). The NL_A_ model can account for CV and HRCC measures, while the DD and NL_P_ models can explain CV values and hub occurrence times.

**Table 1.** Comparison of model performances. Agreement of preferential attachment (PA), duplication–divergence (DD) and nonlinear growth models (NL_A_ or NL_P_) with features of real-world networks. Symbols denote whether hub occurrence time (◻), CV (|), HRCC (−) or all of the previous three (⊞) matched real-world networks.

	PA	DD	NL_A_	NL_P_
protein–protein interactions	|	⊞	+	◫
macaque cortical network	⊞	⊞	+	◫
*C. elegans* neuronal network	+	◫	+	◫
airport flight connections	◻	⊞	+	◫

**Table 2.** Properties of the collected datasets. The number of nodes, number of edges, the average degree *μ*, the maximum degree, the coefficient of variation (CV) and the hub-rich-club coefficient (HRCC) are listed.

	no. nodes	no. edges	*μ*	max(k)	CV	HRCC
protein–protein interactions	1857	9792	10.5	168	1.45	0.22
macaque cortical network	94	2390	50.9	111	0.51	0.88
*C. elegans* neuronal network	279	2990	21.4	137	0.79	0.60
airport flight connections	359	13 460	75.0	314	0.77	0.77

### 3.5. Case study: are alterations of connectivity in preterm-birth related to nonlinear growth?

Finally, we have investigated the explanatory power of the NL model with regard to a recent study [24], where the brain structural connectome in very preterm-born adults was analysed. Counterintuitively, the very preterm brain exhibits a stronger rich-club architecture than the control brain, despite a decrease of white matter resources.

Interestingly, our model can account for this difference. We demonstrate this by simulating the growth of weighted networks exhibiting the rich-club property. Our simulations evolve according to two scenarios. In the control scenario, the network expands nonlinearly as proposed above. Moreover, the connections are weighted, i.e. connections are attributed a scalar value instead of a binary value. This weight (or connection strength) reflects the thickness of fibre tracts in brain networks (i.e. fibre tracts comprising many more fibres than others have higher weights). In our model, we assume that the weight of newly formed connections decreases linearly with developmental time, i.e. the early connections are the strongest. In the pathological scenario, the scalar is additionally decreased when 75% of the network size has been reached (i.e. referring to the time-point of premature birth). Hence, the number of connections is the same in both scenarios; however, the weights of connections developing after preterm birth are decreased in the second scenario (in accordance with the analysis results of Karolis *et al.* [24]).

**Figure 5.**
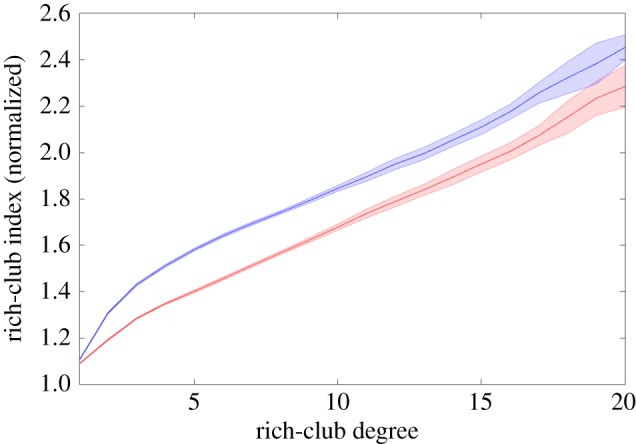
Normalized rich-club indices of control networks (red) and simulated, very preterm brain networks (blue). Shaded areas indicate standard deviation, as obtained from 20 curves per scenario. The *x*-axis indicates the rich club degree, i.e. the cut-off degree defining the rich-club. For the normalization of rich-club indices (*y*-axis), 100 reference networks preserving degree distribution were generated (hence, the number of nodes and node degree distribution do not differ in control, preterm and reference networks). These results demonstrate that the nonlinear growth model is in accordance with observations of increased rich-club organization in the structural connectome of very preterm-born adults (see also fig. 4 of [24]).

For a valid comparison of the two scenarios, the normalized rich-club indices are computed by dividing the rich-club coefficient by the mean rich-club coefficient of 100 reference networks: $\phi (k)/\bar{\phi_{r}(k)}$, where *ϕ*(*k*) and *ϕ*_*r*_(*k*) denote rich-club coefficients for grown and reference networks, respectively, for a given rich-club degree *k*. As shown in figure 5, the pathological networks indeed exhibit a stronger rich-club organization than control networks. The reason is that the simulated premature brain networks have comparably enhanced strength within rich clubs, as the weight factor is decreased for the connections that are formed late during development (i.e. connections of the last 25% nodes). Hence, our model is in agreement with the finding of Karolis *et al.* [24] that there are no topological connectivity differences between the two groups. More information on the growth model for the weighted networks is available in the electronic supplementary material.

## 4. Discussion

We hypothesize that nonlinear growth explains hubs in many networks where nodes do not require detailed knowledge about nodes to which new nodes connect to (e.g. the degree of a node), and where information exchange is local. This is for instance the case for spatial networks where transmitting information over long distances can be prohibitively expensive. For example, during neural development the secretion and detection of diffusable substances are widely utilized processes for conveying critical information. However, the amount and detail of information that can be reliably transmitted in this way is limited. Our model solely requires that a newly added node can potentially form connections with the other nodes, and relies on less information than previous local models [7,25]. Along those lines, axons from one brain area can project to potential target areas based on guidance cues and/or genetically encoded growth mechanisms [26,27]. Hence, our model provides a framework where axonal connections do not need to rely on specific information about these targets, other than the well-established mechanisms required to connect with them. Crucially, the nonlinear growth model does not include space and is complementary to spatial models. As the model’s requirements for the formation of connections are minimal, it is compatible with other models that include Euclidian distance [28] or properties of network connectivity [7].

Our work suggests that real-world networks substantially differ in terms of their hub-related network properties, depending on whether the principle of locality is a determinant of their development. Analyses of other real-world networks could support this hypothesis. Indeed, our model is unable to reproduce hub birth times in citation networks where links to spatially distant researchers nowadays do not involve extra costs (electronic supplementary material, figure S8).

In addition to biological and man-made networks, we particularly propose that nonlinear growth can account for the structural dynamics of neural networks during early (exponential) phases of brain development. In the mammalian brain, a single node could be a region of interest that comprises a large number (e.g. millions) of neurons. In more simple species, such as *C. elegans* or *Drosophila*, a node would correspond to only a few or single neurons. Indeed, studies on brain evolution and development support an exponential increase in the number of brain regions [9] and neurons [29–32]. Interestingly, in contrast with the model in Vértes *et al.* [33], nonlinear growth explains how rich-club organization arises without any access to non-local information. Moreover, our model is in agreement with observations of brain structure following neurodevelopmental disruption after very preterm birth; counterintuitively, rich-club organization is increased in such atypical scenarios [24]. Hence, the nonlinear growth model provides a general and mechanistic understanding of these findings.

The assessment of the growth models is conducted on a topological basis, hence functional differences between individual nodes are not accounted for. Also, we have focused on the development of hub-related network features. In the future, additional growth mechanisms involving secondary mechanisms (removal of connections or node-specific connection formation) could be incorporated to establish small-worldness and modularity [28]. Such additional model complexity could be incorporated to generate networks matching the degree distribution itself, rather than the specific hub-related network measures which were assessed in this work. In addition, we only tested for accelerated growth where the number of new nodes at each step increases: it will be interesting to also test the effects of reductions in the growth rate, meaning scenarios where each step produces fewer nodes than for the previous steps. This aspect highlights that nonlinear growth, as linear growth, is limited by the maximum size of the network that can or should be sustained.

Our model for hub formation does not imply a scale-free degree distribution (electronic supplementary material, figure S9), which is an independent feature for some networks [17,34]. Hence, our model is well suited for scenarios where such scale-freeness is not established but hubs are nonetheless known to exist [35]. We emphasize that the nonlinear growth principle is complementary to many mechanistic explanations for how neural connectivity arises during development [17,36] and evolution [37].

Overall, nonlinear growth relies solely on locally available information (e.g. nodes do not require knowledge about the degree of connection partners), and so can provide a baseline benchmark for modelling network evolution that contains phases of exponential growth. We have demonstrated its explanatory power with respect to hub-related network features, in comparison with well-established classical models. Not all growth models that can generate hubs have been investigated in this study, because such an extensive study would entail an extremely high number of parameter optimizations. However, to the best of our knowledge, all alternative models assume that connecting nodes have access to more information than the nonlinear growth model relies upon (e.g. [38–40]). Therefore, the nonlinear growth model, either using relative or absolute numbers of edges from each new node, provides a general principle for hub development in certain spatially embedded and/or biological networks. Furthermore, we propose that the nonlinear growth model could be applied for the efficient design of spatial networks (e.g. power grids and supply networks), where robustness and information integration are crucial properties for network function [22].

## Supplementary Material

ESM.pdf: Information on materials, methods and parameters.

## Data accessibility

Our data are deposited at the Dryad Digital Repository: http://dx.doi.org/10.5061/dryad.6h8pm [41].
