# Supplementary material for: Nonlinear growth: an origin of hub organization in complex networks
Source: R Soc Open Sci. 2017 Mar 22;4(3):160691. doi: 10.1098/rsos.160691 (PMC5383813; doi:10.1098/rsos.160691)
Supplement: ESM.pdf: Information on materials, methods and parameters. [file rsos160691supp1.pdf]

Electronic supplementary material for ”Nonlinear growth: An origin of network hubs”

Network datasets description

Protein-protein interactions (PPI) dataset. The yeast PPI dataset with estimated protein ages was obtained based on the high-throughput (HTP) dataset from [1] which the authors collected from several publications. After exclusion of proteins without assignment of age category and node degree of zero, the interaction network comprises $1\,857$ different proteins and $9\,738$ interactions. The highest degree node has 168 connections. The authors have also estimated the age groups of yeast proteins by computing the taxonomic distributions of Pfam domains for different microorganisms. Based on the phylogenetic tree of these species and the assigned Pfam domains of the proteins, they derived 4 discrete age categories for the yeast protein network. For our purposes, we used 3 age categories and merged the last two originally obtained age categories. Since protein interactions are always bidirectional, the target number of edges for the optimization algorithm was chosen half the number of bidirectional interactions $(4\,869)$, such that the final connectivity matrix can be obtained by summation of the model matrix and its transpose.

Macaque dataset. This dataset of the macaque inter-areal brain connectivity within one hemisphere stems from the CoCoMac database [2] and was down-

loaded from http://www.dynamic-connectome.org/?page_id=25. The connectivity matrix has 94 nodes and 2 390 edges. The maximal node degree is 111. The assignment of temporal maturation was done using MRI-based assessment of maturational trajectories [3], where the volume of brain regions from rhesus monkeys was tracked over time (between 10 and 64 months of age). Assignment of temporal maturation for the macaque dataset was done according to [3]. The authors derive 3 distinct types of trajectories: no change, linear increase and biphasic. We assigned the early maturation bin to the regions where no change was found (because they reach their maximum volume early on during development), intermediate maturation time to biphasic (because they reach their peak volume after the first group), and the late-maturing to the linearly increasing group. In humans, it has been shown that many different maturation factors such as peak cortical thickness, mature gray matter volume, synaptic density etc. have consistent temporal sequences [4], and so we believe that gray matter volume trajectories are a valid measure for maturation assessment. The dataset is available from the Dryad Digital Repository: http://dx.doi.org/10.5061/dryad.6h8pm.

C. elegans dataset. The *C. elegans* network was obtained from http://www.wormatlas.org/neuronalwiring.html#Connectivitydata [5]. The connectivity including chemical synapses and gap-junctions transmitting electrophysiogical activity was extracted. Neuronal birth times were downloaded from http://www.dynamic-connectome.org/?page_id=25 (data stems from [6, 7]). The final network consists of 279 neurons, connected by 2 990 (directed)

connections, the maximal node degree being 137. Since each connection can have multiple synapses, only the binary value (connection formed / not formed) was used for computing the network statistics/measures.

AIR dataset. The connectivity of the AIR dataset was obtained from http://www.dynamic-connectome.org/?page_id=25. Only symmetric connections were selected (91.11% of all connections). Airport construction dates were collected using a Matlab script that searched through German and English Wikipedia sites. Based on this process, 359 airports with construction dates were collected. The network comprises $13\,460$ flight connections, and the node with maximal degree (Frankfurt Airport) participates in 314 (in- and outgoing) connections. Collection of the airport construction dates was done using the IATA identifiers in the web search. The hereby found websites were temporarily stored, and the construction dates were obtained if available directly on the site. If the web crawler did not find these information indicated directly, it searched for keywords such as 'opened','built','inaugurated'. The earliest of these dates was then used as the estimated construction date. Few examples were assigned by person. The dataset is available from the Dryad Digital Repository: http://dx.doi.org/10.5061/dryad.6h8pm.

High-energy physics theory (HEPTH) dataset. The connectivity and time-based information of the HEPTH dataset were obtained from http://www.snap.stanford.edu/data/cit-HepTh.html. The dataset describes the citation graph of the e-print arXiv in the high-energy physics theory field. The time span is from January 1993 to April 2003 (124 months). The final network

(after discarding for nodes with zero node degree or where no time information was available) consists of $10\,732$ nodes and $81\,088$ edges. The maximal node degree is 668.

Methods

Implementation of Models

In addition to the description of the main models in the manuscript, the Matlab scripts of the nonlinear growth model are available from the Dryad Digital Repository: `http://dx.doi.org/10.5061/dryad.6h8pm`.

Alternative Implementations of Models

An alternative version of the PA model as described in [8, 9] has been implemented (generalized PA or GPA). In this scenario, the baseline probability decreases with time: $p_i = p_1 \cdot \frac{k_i}{\sum\limits_j k_j} + \frac{p_2}{1+t}$. Also, we have implemented a version of the PA model where the absolute number of outgoing projections is limited. As in the $\mathrm{NL_A}$ model, this absolute projection number is computed by drawing random values from a normal distribution with mean a and standard deviation $a/2$, where a indicates the second parameter of the model. This value is discretized using the Matlab round.m function, and bounded between 0 and the current number of nodes in the network. The GPA model is in contrast to the PA model or the $\mathrm{NL_P}$ model, where the number of outgoing projections per node is not bounded (new nodes connect to any node with equal probability p). The network measures as

produced by the GPA and $\mathrm{PA_A}$ models are shown in Supplemental Fig. S7. Overall, the GPA and $\mathrm{PA_A}$ models perform similar to the PA implementation (Table S1).

Visualization

The visualization of the macaque brain in Fig. 1 was created using the Caret software [10] (`http://brainvis.wustl.edu/wiki/index.php/Caret:About`). The dataset of a left macaque hemisphere was downloaded from the Surface Management Systems DataBase (`http://sumsdb.wustl.edu:8081/sums/index.jsp`).

Parameters

The model parameters we used to generate the CV values (after optimization) are listed in Supplemental Table S2. Model parameters for the HRCC values and the trajectories of hub occurrence (after optimization) are listed in Supplemental Tables S3 and S4, respectively. Corresponding model parameters for the alternative PA models are shown in Supplemental Table S5.

The growth of the weighted rich-club networks analyzed in Fig. 5 was done using the NL_A model. For both scenarios (control and pathological), the network size was 200, and parameters $a = 4.5, x = 2.5$ were used. A multiplicative factor of $\frac{1.5}{t}$ was used to set the connection strength of the connections created at time step t during network growth (initially $t = 1$). For the pathological networks, the multiplicative factor changed to $\frac{0.1}{t}$ after 75% of the nodes were born.

CV value as an indicator of outliers

We have used the CV value as an indicator of outliers in the degree distributions. Outliers effect the mean more compared to the median, such that the ratio of mean over median is also an indicator for hubs. Supplemental Fig. S1 shows that the CV value indeed correlates with the ratio of mean over median.

Number of edges in model networks

As mentioned before, CV and HRCC values were optimized conditional upon the number of edges matching the numbers of the collected datasets. Supplemental Fig. S3A shows the discrepancy of the model-generated number of edges compared to the target numbers for the CV-targeted simulations. Accordingly, Supplemental Fig. S3B,C show the distribution of the relative errors for the simulations where the HRCC value and the birth times of hubs, respectively, were optimized.

Node degrees and birth times

Supplemental Fig. S4 shows the node degree across the different birth times of the nodes during network development. For the PPI, macaque, *C. elegans* and AIR datasets, the earliest time bin predominantly comprises hubs.

Early-born nodes have high in-degree

While early nodes finish the formation of connections to other nodes, they can still receive incoming connections from nodes that are added later. The earlier a node is established, the longer it can receive connections from other nodes. We

therefore expect that early nodes receive a higher total number of incoming connections than later-born nodes. This is indeed the case for the *C. elegans* network (Supplemental Fig. S5A). Correlations involving the ratio of in- vs out-degree were not statistically significant (Supplemental Fig. S5B,C). As the correlation between developmental time and in-degree can be accounted for by all growth models, it cannot be used to distinguish between growth types.

Assessment of complementary network measures

Also complementary network features that are not directly related to hubs have been analyzed. Supplemental Fig. S6 and S7 show the features, together with the (optimized) CV and HRCC values. In the future, additional mechanisms could be incorporated in the nonlinear growth model, in order to produce networks that account also for network features that do not directly relate to hubs.

Model of impact of neurodevelopmental disruption on rich-club architecture

We implemented the development of weighted networks, to analyse differences in rich-club organization of such networks under control conditions and under pathological development. 20 networks consisting of 200 nodes were grown based on the NL_A model, using the model parameters $x = 2.5$ and $p = 4.5$. In addition to the formation of new nodes and connections at each time step, the newly formed connections were attributed with continuous weights, reflecting the strength of connections. The weights are large at the beginning, and decay linearly with time: $W(t) = \mu \cdot r \cdot (1 + t_{max} - t)$, where t is the current time step, t_{max} is the to-

tal number of time steps it takes until the network is generated, $\mu = 1.5$ is a model variable, and r is a uniformly distributed random variable in the interval [0.5,1.5]. The growth of pathological networks (reflecting the rich-club organizational features of very preterm brain networks [11]) was also simulated using the NL_A model and the same parameters. However, the weight of newly formed connections changed after maturation of 75% of all nodes (i.e. 150 nodes) to $W(t) = \mu_{pathol} \cdot r \cdot (1 + t_{max} - t)$, with $\mu_{pathol} = 0.1$. Hence, the last 25% of nodes in the early preterm networks formed significantly weaker connections than those of the control networks. The rich-club coefficient was computed by normalizing by the rich-club coefficients of each network based on 100 degree-preserving reference networks. The standard deviation indicated in Fig. 5 of the manuscript is computed based on the 20 normalized rich-club coefficient curves.

Results for citation network dataset (HEPTH)

In contrast to the PPI, macaque, *C. elegans* and AIR networks, hubs in the high-energy physics citation network (HEPTH) arise mostly later during network development. This may be due to changes in funding policies, as well as very early publications not being in the mainstream research domain (i.e. global factors). None of the models could account for the hub occurrence trajectories in the HEPTH dataset. Supplemental Fig. S8 shows one optimization result in the case of the $\mathrm{NL_P}$ model.

Degree distribution of collected networks

The degree distributions of the collected datasets are shown in Supplemental Fig. S9. The distributions exhibit a variety of behaviours, and often do not follow a scale-free distribution (e.g. for the macaque interareal connections).

References

[1] Kim WK, Marcotte EM. Age-dependent evolution of the yeast protein interaction network suggests a limited role of gene duplication and divergence. PLoS Comput Biol. 2008;4(11):e1000232.

[2] Kötter R. Online retrieval, processing, and visualization of primate connectivity data from the CoCoMac database. Neuroinformatics. 2004;2(2):127–144.

[3] Knickmeyer RC, Styner M, Short SJ, Lubach GR, Kang C, Hamer R, et al. Maturational trajectories of cortical brain development through the pubertal transition: unique species and sex differences in the monkey revealed through structural magnetic resonance imaging. Cereb Cortex. 2010;20(5):1053–1063.

[4] Hill J, Inder T, Neil J, Dierker D, Harwell J, Van Essen D. Similar patterns of cortical expansion during human development and evolution. Proc Natl Acad Sci. 2010;107(29):13135–13140.

[5] Varshney LR, Chen BL, Paniagua E, Hall DH, Chklovskii DB. Structural properties of the *Caenorhabditis elegans* neuronal network. PLoS Comput Biol. 2011;7(2):e1001066.

[6] Sulston J, Horvitz H. Post-embryonic cell lineages of the nematode, *Caenorhabditis elegans*. Dev Biol. 1977;56(1):110–156.

[7] Sulston J. The embryonic cell lineage of the nematode *Caenorhabditis elegans*. Dev Biol. 1983;100(1):64–119.

[8] Dorogovtsev SN, Mendes JFF, Samukhin AN. Structure of growing networks with preferential linking. Phys Rev Lett. 2000;85(21):4633–4636.

[9] Pennock DM, Flake GW, Lawrence S, Glover EJ, Giles CL. Winners don't take all: Characterizing the competition for links on the web. Proc Natl Acad Sci. 2002;99(8):5207–5211.

[10] Van Essen DC, Drury HA, Dickson J, Harwell J, Hanlon D, Anderson CH. An integrated software suite for surface-based analyses of cerebral cortex. J Am Med Inform Assoc. 2001;8(5):443–459.

[11] Karolis VR, Froudist-Walsh S, Brittain PJ, Kroll J, Ball G, Edwards AD, et al. Reinforcement of the Brain's Rich-Club Architecture Following Early Neurodevelopmental Disruption Caused by Very Preterm Birth. Cereb Cortex. 2016;p. bhv305.

[12] Achard S, Salvador R, Whitcher B, Suckling J, Bullmore E. A resilient, low-frequency, small-world human brain functional network with highly connected association cortical hubs. J Neurosci. 2006;26(1):63–72.

[13] Sporns O, Honey CJ, Kötter R. Identification and classification of hubs in brain networks. PLoS ONE. 2007;2(10):e1049.

[14] Hase T, Tanaka H, Suzuki Y, Nakagawa S, Kitano H. Structure of protein interaction networks and their implications on drug design. PLoS Comput Biol. 2009;5(10):e1000550.

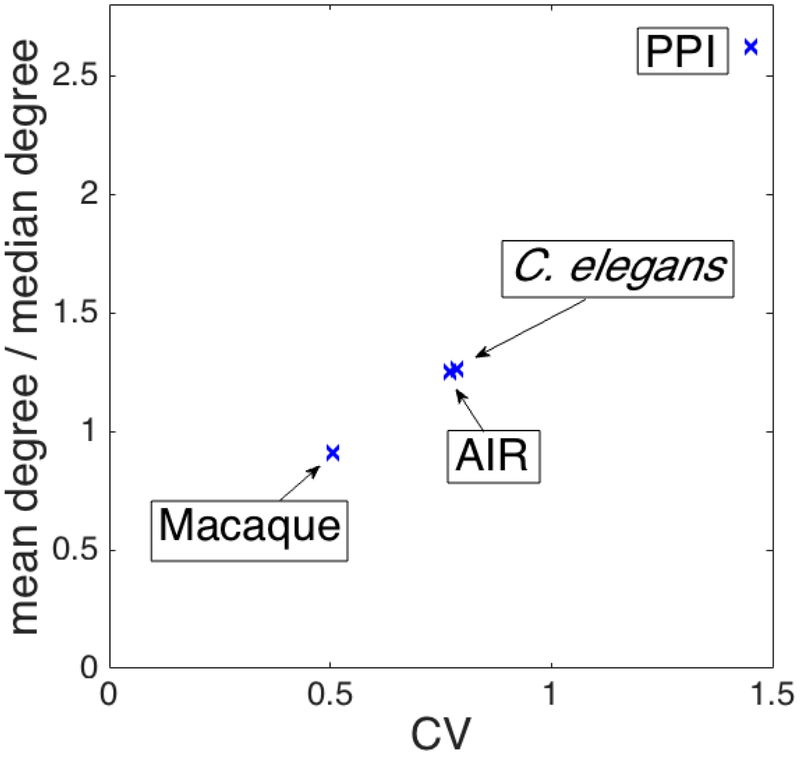

Figure S1: The CV value serves as an indicator of outliers in the degree distribution. The degree distributions' CV values are plotted against the ratio of mean *vs.* median. Hubs lead to higher mean values compared to medians, which correlates positively with higher CV values.

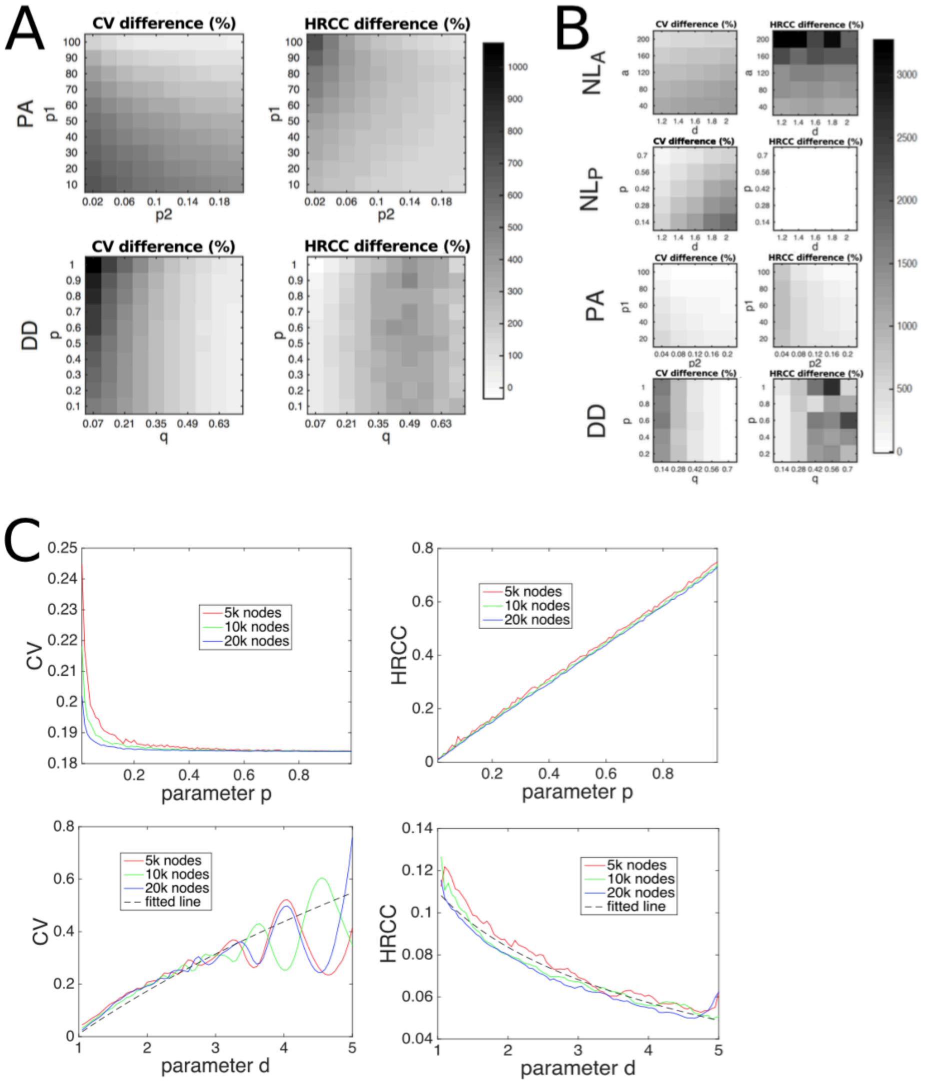

Figure S2: *(A)* PA and DD models produce hub-related complex network properties. Degree variation (CV) and mean rich-club coefficient between hubs (HRCC) of the two nonlinear growth models, across different model parameters. The relative deviations from regular, random networks of matched size and edge density are displayed as grayscale (see colorbar). *(B)* Variation of CV and HRCC values for large networks of 10'000 nodes, for the PA, DD, NL_A and NL_P models. The results confirm a strong deviation of characteristic network properties from those of random, regular networks across different parameter ranges. The number of parameter changes was decreased because of the higher computer resource demands. *(C)* Parameter dependencies of the CV and HRCC values in the NL_P model, across different network sizes. Variation of parameter p (top) with $d = 2$ generates an exponentially converging curve for the CV value, while the HRCC increases linearly. Variation of parameter d (bottom) with $p = 0.1$ produces CV and HRCC values that can be approximated with $log(0.83 + d \cdot 0.18)$ and $0.11 - 0.038 \cdot log(d)$, respectively. For clearer comparison, the data curves in the bottom plot were smoothed using the moving average method.

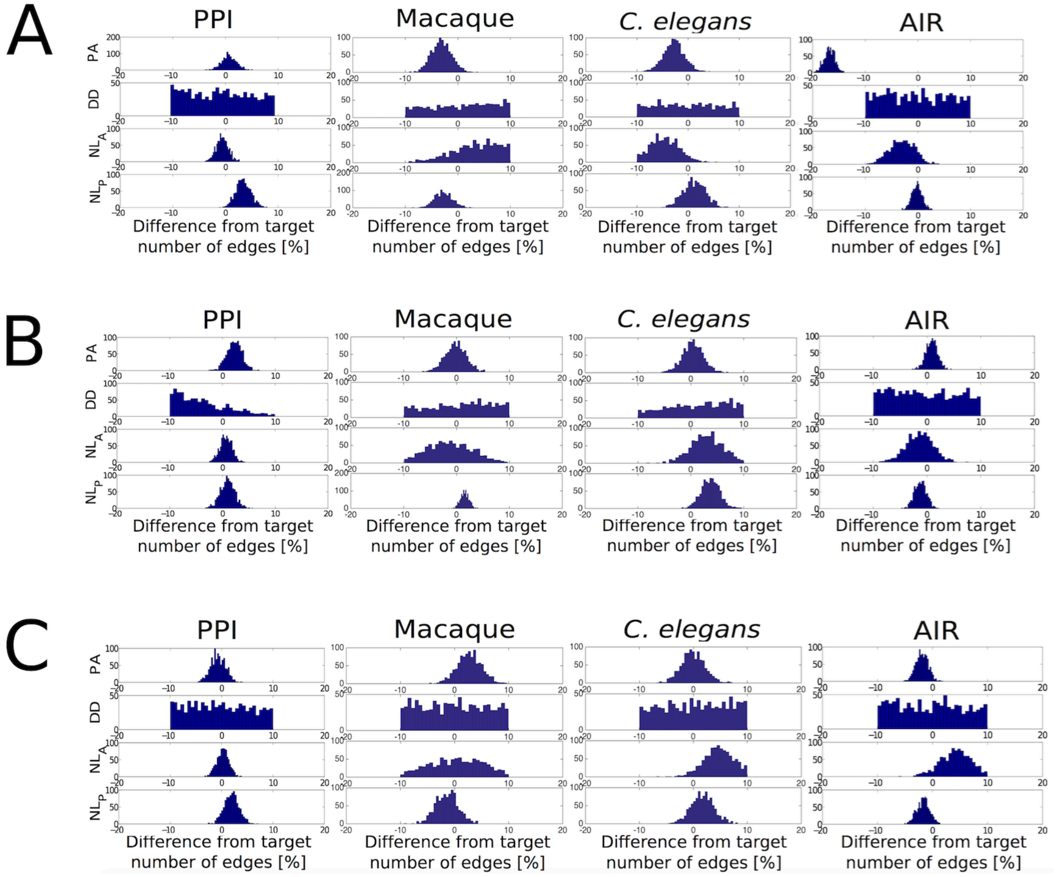

Figure S3: Deviations of the number of connections from the measured number. Relative deviations of the number of connections in the model networks from the measured number. The error distribution is shown for each of the models, datasets and optimized network measures. *(A)* The growth models were optimized for yielding the CV values of the PPI, macaque, *C. elegans* and AIR datasets. *(B)* The growth models were optimized for yielding the HRCC values of the PPI, macaque, *C. elegans* and AIR datasets. *(C)* The growth models were optimized for yielding the hub occurrences in the different developmental time bins of the PPI, macaque, *C. elegans* and AIR datasets.

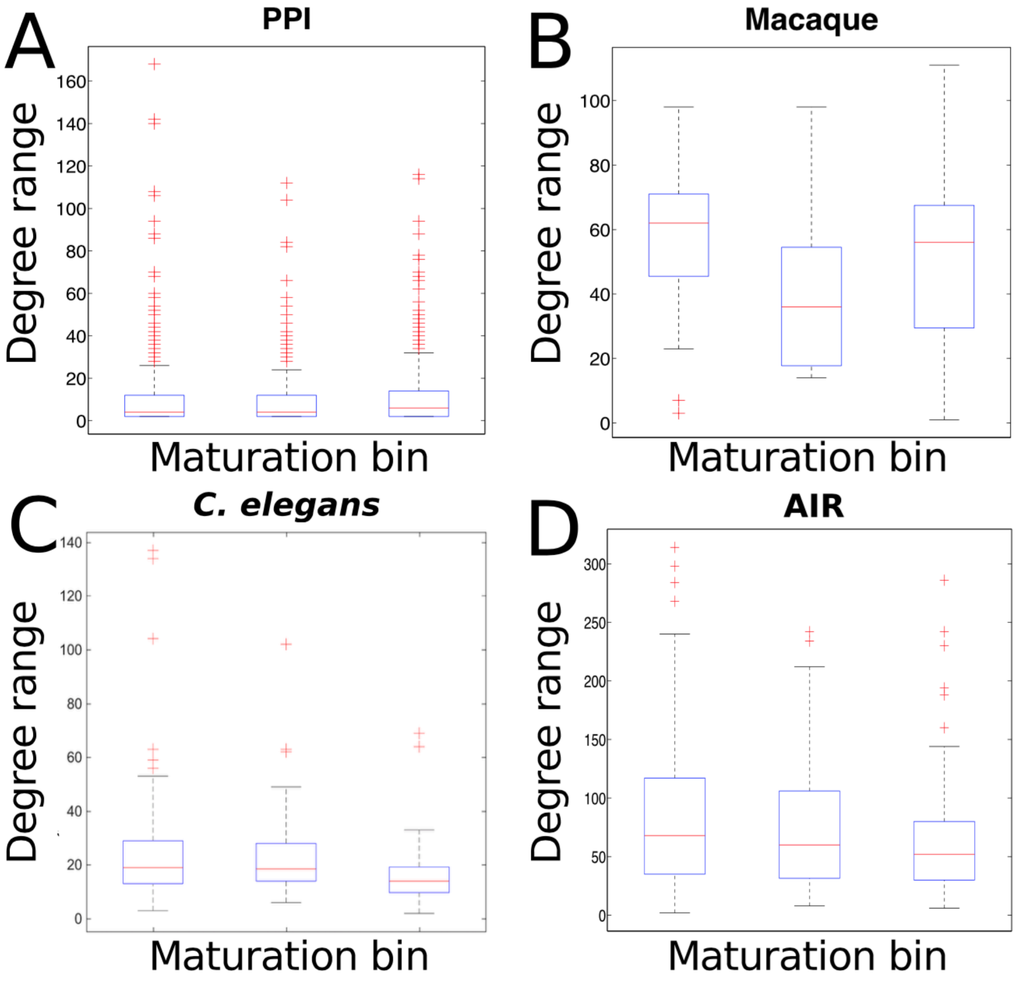

Figure S4: Node degrees across the maturation bins. The binning was done according to the discrete maturation/age groups (PPI *(A)* and macaque *(B)* datasets), or by regularly dividing the total developmental time into 3 time segments (*C. elegans (C)* and AIR *(D)* datasets).

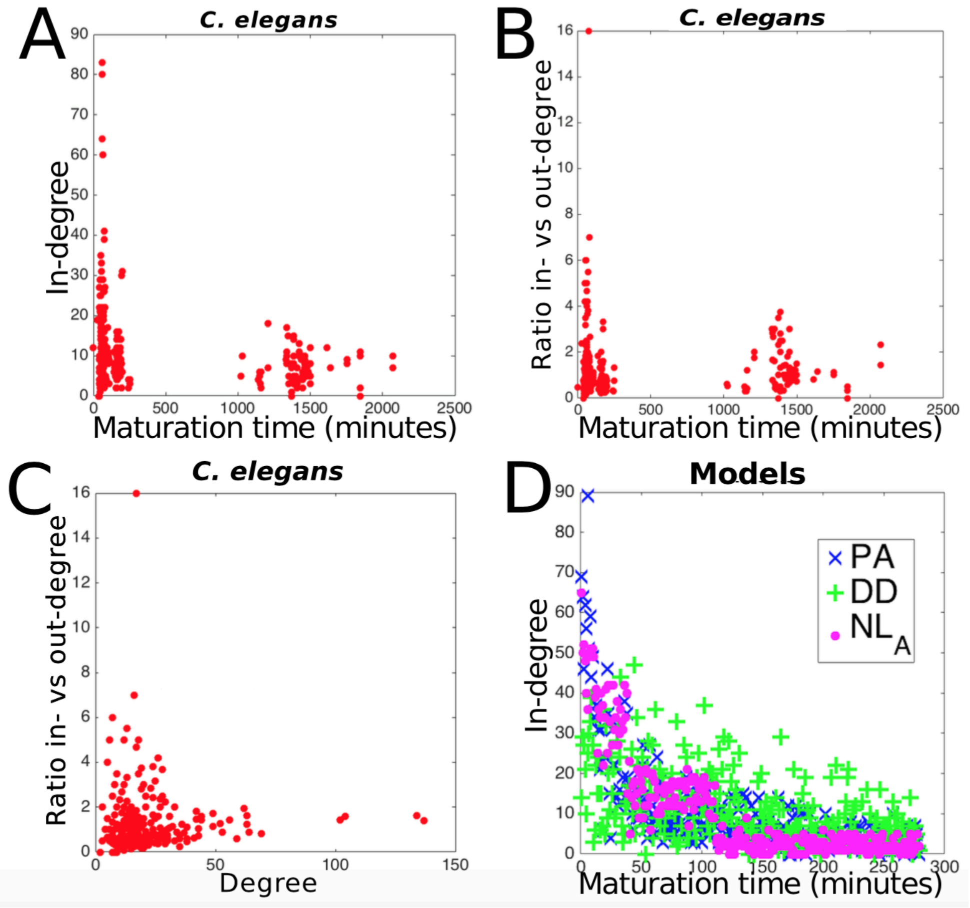

Figure S5: Developmental maturation and degree statistics. *(A)* Correlation of nodal in-degree with developmental time in the *C. elegans* ($R = -0.2205$, $p = 0.0002$). *(B,C)* Correlation of nodal in- *vs.* out-degree ratio ($\frac{d_{in}}{d_{out}}$) with developmental time (B) and degree (C). (A-B) exhibit negative correlations, with only (A) being statistically significant. Since many of the areas in the macaque were not tested in both directions, this figure includes only the *C. elegans* connectivity. *(D)* Comparison of the model-generated data. The model parameters were adapted such that the generated networks matched the same benchmark network statistics (279 nodes and 2990 edges). The model-generated samples exhibit a statistically significant correlation (R=-0.6437 for PA, R=0.5215 for DD, R=0.7815 for $\mathrm{NL_A}$ and $p < 10e - 4$ for each of them) demonstrate that this relationship can be accounted for by all 3 models.

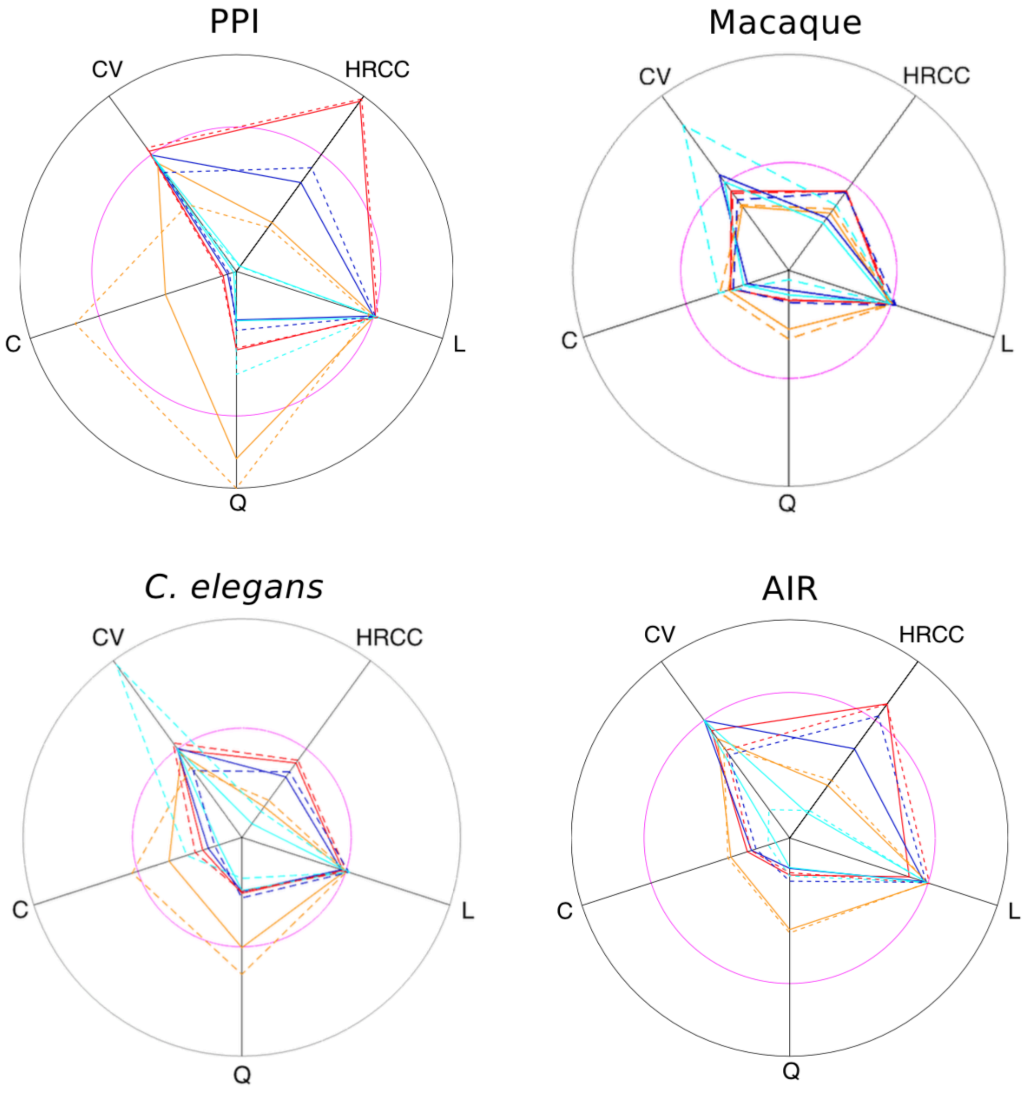

Figure S6: Comparison of model performances with respect to different network measures, after parameter optimization for the CV (solid lines) and HRCC (dashed lines) values. Axes indicate the ratio of a model-generated network measure *vs.* the dataset's measure: $\frac{\phi_{model}}{\phi_{data}}$. ϕ_{model} is the mean of the measures computed from 1000 model-generated networks. Magenta circles indicate a ratio of 1. The models (PA, red; DD, orange; NL_A, cyan; NL_P, blue) are compared with respect to the optimized measures, as well as complementary measures (clustering coefficient C; modularity index Q; characteristic path length L).

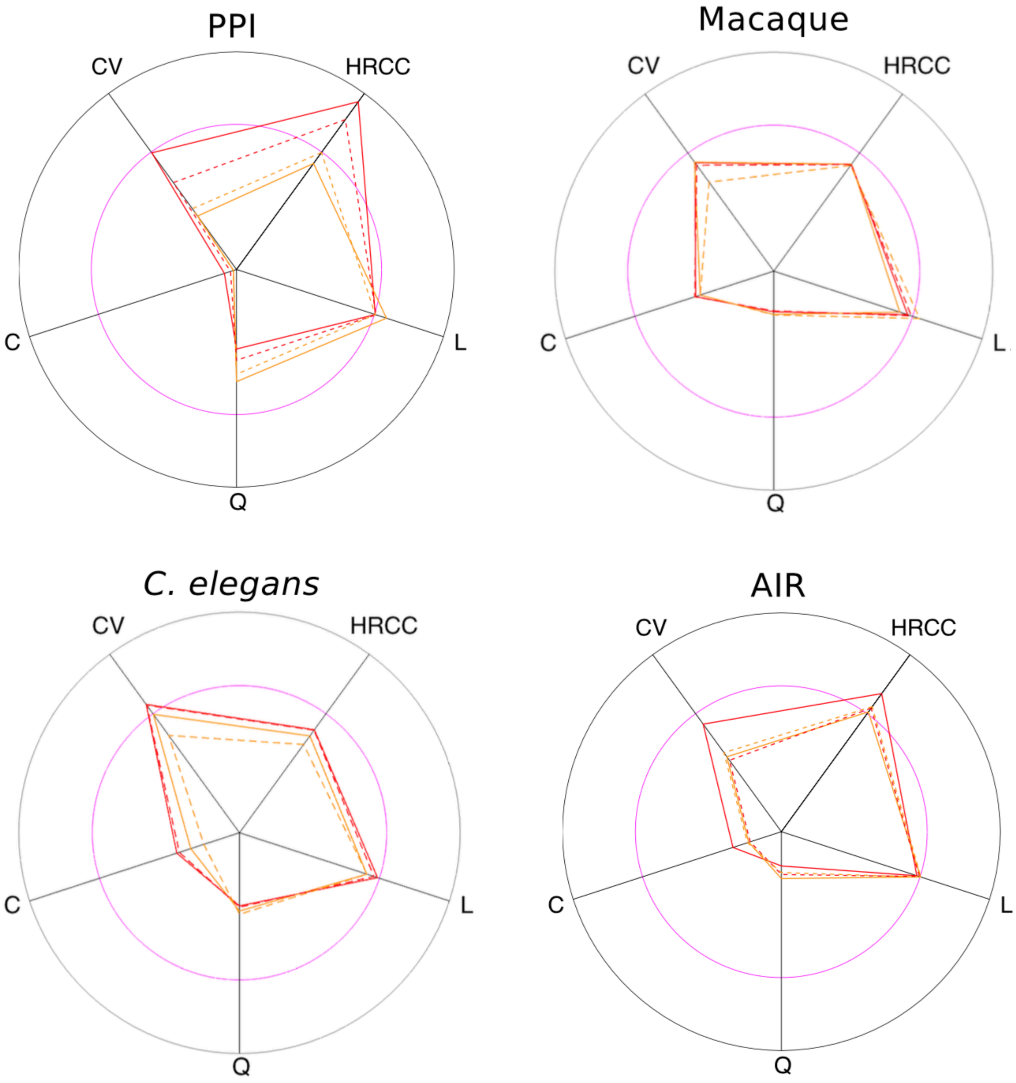

Figure S7: Comparison of model performances with respect to different network measures, after parameter optimization for the CV (solid lines) and HRCC (dashed lines) values. Axes indicate the ratio of a model-generated network measure *vs.* the dataset's measure: $\frac{\phi_{model}}{\phi_{data}}$. ϕ_{model} is the mean of the measures computed from 1000 model-generated networks. Magenta circles indicate a ratio of 1. The models (GPA, red; PA_A, orange) were compared with respect to the optimized measures, as well as the complementary measures (clustering coefficient C; modularity index Q; characteristic path length L).

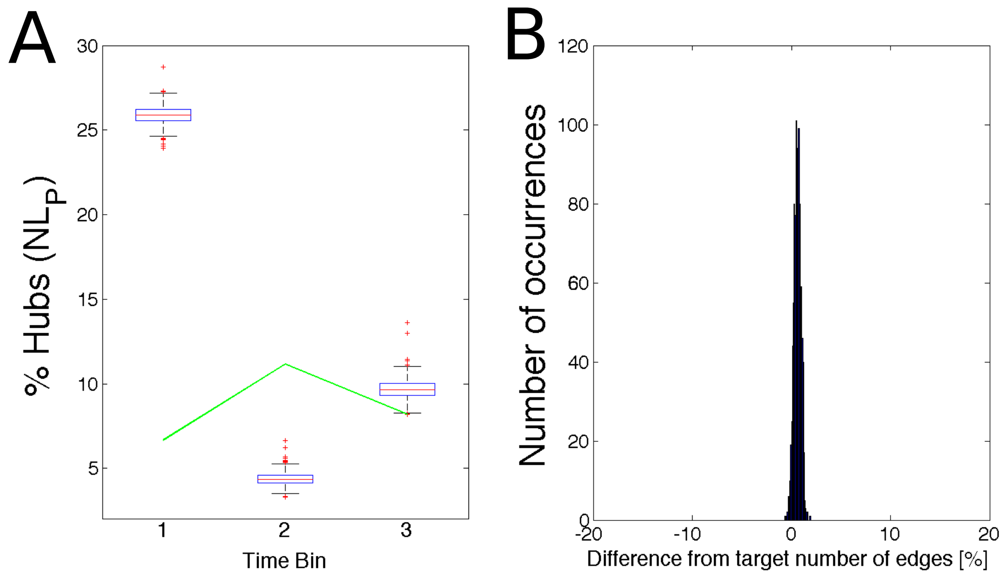

Figure S8: Hub occurrences of HEPTH dataset. *(A)* Distribution of hub occurrences in the different time bins for an example parameter set in the $\mathrm{NL_P}$ model, after optimization for the HEPTH dataset. The target trajectory (green) could not be matched. *(B)* Deviation of the number of edges from the dataset for the sample networks in (A). Overall, none of the models were able to account for the HEPTH hub occurrences, which could be due to influences not captured in these models (e.g. funding policies or economical conditions), and the non-local information exchange in the establishment of new connections. In particular, there is an increase in the number of hubs from the first to the second time bin in the HEPTH dataset (in contrast to the PPI, macaque, *C. elegans* and AIR datasets).

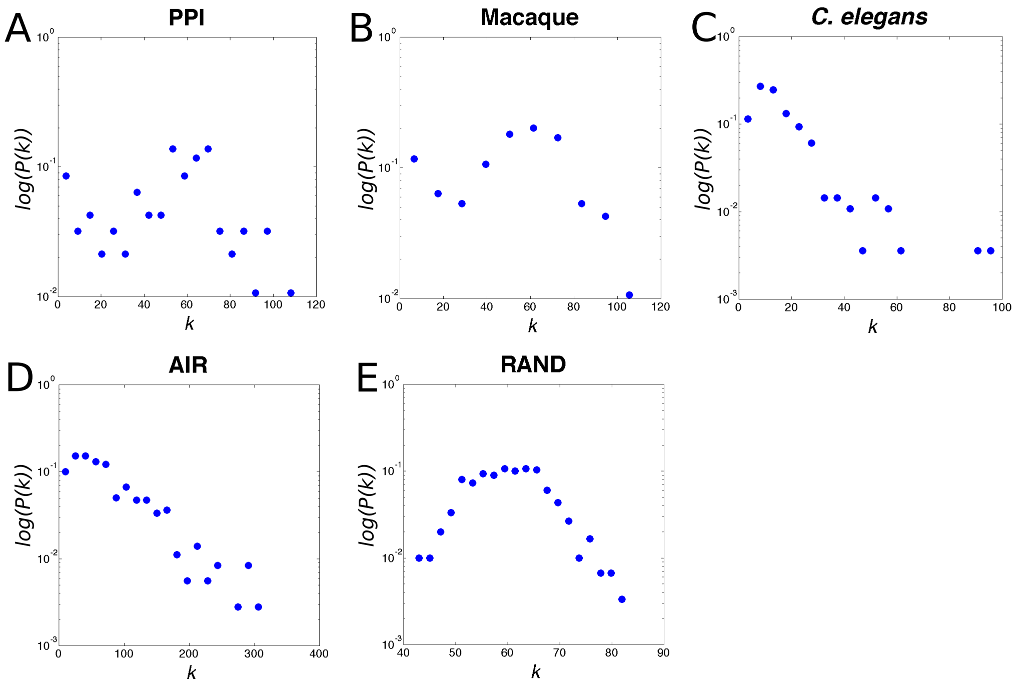

Figure S9: Semi-log degree distributions of datasets *(A-D)* Semi-log plots of the degree distributions in the different datasets. The distributions exhibit characteristics that are different from regular, random networks *(E)*. Due to a low number of network nodes, it is unclear for many network types whether they are scale-free or not. Along these lines, many complex networks do not follow a scale-free degree distribution, but comprise hubs [12, 13, 14].

Supplementary Tables

Table S1: Agreement of two alternative models of the PA model (GPA and $\mathrm{PA_A}$ models) with features of real-world networks. Symbols denote whether the hub occurrence time ($\square$), the CV value ($|$), the HRCC value ($-$) or all ($\boxplus$) matched the real-world networks.

	GPA	$\mathrm{PA_A}$
Protein-protein interactions	$+$	$+$
Macaque cortical network	$+$	$+$
C. elegans neuronal network	$+$	$+$
Airport flight connections	$-$	$-$

Table S2: Parameters of the growth models. Simulated annealing was used for optimizing the CV value of the degree distribution, conditional on the number of nodes and edges as in the respective dataset.

	PA	DD	$\mathrm{NL_A}$	$\mathrm{NL_P}$
Protein-protein interactions	$p_1 = 2.568$	$p = 0.1731$	$a = 2.6191$	$p = 0.0162$
	$p_2 = 9.512 \cdot 10^{-5}$	$q = 0.4452$	$d = 10.6348$	$d = 40.9272$
Macaque cortical network	$p_1 = 17.2112$	$p = 0.6709$	$a = 40.9130$	$p = 0.6338$
	$p_2 = 0.0019$	$q = 0.1640$	$d = 7.6516$	$d = 3.9719$
C. elegans neuronal network	$p_1 = 7.2712$	$p = 0.3305$	$a = 7.9735$	$p = 0.1566$
	$p_2 = 0.0058$	$q = 0.3075$	$d = 2.9266$	$d = 6.0108$
Airport flight connections	$p_1 = 15.8943$	$p = 0.3216$	$a = 19.4654$	$p = 0.3057$
	$p_2 = 3.766 \cdot 10^{-4}$	$q = 0.1968$	$d = 4.9503$	$d = 8.0961$

Table S3: Parameters of the growth models. Simulated annealing was used for optimizing the HRCC value of the degree distribution, conditional on the same number of nodes and edges as in the respective dataset.

	PA	DD	$\mathrm{NL_A}$	$\mathrm{NL_P}$
Protein-protein interactions	$p_1 = 2.6991$	$p = 0.1746$	$a = 2.6505$	$p = 0.0056$
	$p_2 = 0.0000$	$q = 0.4334$	$d = 6.6798$	$d = 10.5056$
Macaque cortical network	$p_1 = 17.4465$	$p = 0.8018$	$a = 17.8648$	$p = 0.9997$
	$p_2 = 0.0082$	$q = 0.1435$	$d = 1.0936$	$d = 8.7224$
C. elegans neuronal network	$p_1 = 8.2191$	$p = 0.6765$	$a = 8.2279$	$p = 0.3780$
	$p_2 = 0.0011$	$q = 0.3252$	$d = 1.2045$	$d = 15.9941$
Airport flight connections	$p_1 = 15.8585$	$p = 0.4056$	$a = 18.6207$	$p = 0.1248$
	$p_2 = 0.0192$	$q = 0.2239$	$d = 1.2476$	$d = 1.5087$

Table S4: Parameters of the growth models. Simulated annealing was used for optimizing the occurrence of hubs across 3 developmental time bins, conditional on the same number of nodes and edges as in the respective dataset.

	PA	DD	NL_A	NL_P
Protein-protein interactions	$p_1 = 2.6227$	$p = 0.0200$	$a = 2.6218$	$p = 0.0053$
	$p_2 = 0.000$	$q = 0.3891$	$d = 1.7294$	$d = 10.0041$
Macaque cortical network	$p_1 = 1.9941$	$p = 0.0014$	$a = 27.2809$	$p = 0.4406$
	$p_2 = 0.3148$	$q = 0.056$	$d = 6.4706$	$d = 1.8349$
C. elegans neuronal network	$p_1 = 2.1706$	$p = 0.3256$	$a = 9.2254$	$p = 0.1092$
	$p_2 = 0.0434$	$q = 0.1683$	$d = 9.7218$	$d = 11.6757$
Airport flight connections	$p_1 = 1.0953$	$p = 0.2697$	$a = 24.3812$	$p = 0.1087$
	$p_2 = 0.0966$	$q = 0.2101$	$d = 10.3919$	$d = 1.1300$

Table S5: Parameters of the alternative GPA and PA_A growth models. Simulated annealing was used for optimizing the CV values, HRCC measures and and hub occurrence trajectories. The model networks are conditional on the same number of nodes and edges as in the respective dataset.

	GPA	PA_A
Protein-protein interactions (CV)	$p_1 = 2.5103$	$p_1 = 0.3107$
	$p_2 = 0.1279$	$a = 2.5322$
Protein-protein interactions (HRCC)	$p_1 = 1.8685$	$p_1 = 0.8223$
	$p_2 = 0.7905$	$a = 1.8115$
Protein-protein interactions (trajectories)	$p_1 = 2.5407$	$p_1 = 0.2646$
	$p_2 = 0.0887$	$a = 2.5636$
Macaque cortical network (CV)	$p_1 = 17.1754$	$p_1 = 14.7528$
	$p_2 = 0.0208$	$a = 0.4790$
Macaque cortical network (HRCC)	$p_1 = 17.4366$	$p_1 = 3.1486$
	$p_2 = 0.2910$	$a = 16.5948$
Macaque cortical network (trajectories)	$p_1 = 0.1011$	$p_1 = 0.1886$
	$p_2 = 17.9677$	$a = 17.4821$
C. elegans neuronal network (CV)	$p_1 = 8.0746$	$p_1 = 6.1938$
	$p_2 = 0.1499$	$a = 1.0944$
C. elegans neuronal network (HRCC)	$p_1 = 7.6857$	$p_1 = 3.7240$
	$p_2 = 0.3297$	$a = 3.5997$
C. elegans neuronal network (trajectories)	$p_1 = 0.1198$	$p_1 = 0.2029$
	$p_2 = 8.3685$	$a = 7.5947$
Airport flight connections (CV)	$p_1 = 18.6910$	$p_1 = 0.0328$
	$p_2 = 0.1422$	$a = 19.3568$
Airport flight connections (HRCC)	$p_1 = 2.4621$	$p_1 = 5.7414$
	$p_2 = 16.8702$	$a = 13.6785$
Airport flight connections (trajectories)	$p_1 = 16.9038$	$p_1 = 0.1396$
	$p_2 = 2.4284$	$a = 19.1629$
